# Cross-Sectional Study of Serum Galectin-3 Levels in Patients with Type 2 Diabetes and Colorectal Polyps

**DOI:** 10.3390/ijms26167662

**Published:** 2025-08-08

**Authors:** Monika Storman, Adam Przybyłkowski, Leszek Czupryniak

**Affiliations:** 1 Department of Internal Medicine, Pulmonary Diseases and Allergy, Medical University of Warsaw, Banacha 1a, 02-304 Warsaw, Poland; 2 Department of Gastroenterology and Internal Medicine, Medical University of Warsaw, Banacha 1a, 02-304 Warsaw, Poland; adam.przybylkowski@wum.edu.pl; 3 Department of Diabetology and Internal Medicine, Medical University of Warsaw, Banacha 1a, 02-304 Warsaw, Poland

**Keywords:** type 2 diabetes mellitus, galectin-3, colorectal polyps, metabolic factor

## Abstract

Galectin-3 (Gal-3) secreted by activated macrophages is involved in inflammation, fibrosis, and tumorigenesis. It is considered a potential biomarker and therapeutic target. This study assessed the association between serum Gal-3, type 2 diabetes (T2D), and colorectal polyps (CRPs). In this cross-sectional study, 80 non-cancer patients undergoing colonoscopy were divided into four subgroups based on T2D and CRP status. Serum Gal-3 and metabolic parameters were measured. All patients’ mean serum Gal-3 level was 13.63 ng/mL. Gal-3 levels were significantly higher in T2D+ than in the T2D− group (14.93 ng/mL, *p* = 0.02). Gal-3 concentration correlated significantly with age (rho = 0.281; *p* = 0.012), gender (rho = 0.220; *p* = 0.049), serum peptide C levels (rho = 0.957; *p* = 0.006), and serum IGF-1 levels (rho = −0.417; *p* < 0.001) in all patients, and for patients T2D-, it also correlated significantly with fasting plasma glucose levels (rho = −0.406; *p* = 0.009). A logistic regression analysis of the risk of polyps was conducted (CRP+ vs. CRP−) considering factors such as gender, age, body weight, waist circumference, T2D, HOMA-IR, insulin, API, IGF-1, total cholesterol, and Gal-3. Gal-3 serum was shown to be a strong independent predictor of CRPs regardless of the presence of T2D+ (*p* = 0.031). Gal-3 may correlate with the development of CRPs and might be a candidate biomarker of CRPs/cancer development.

## 1. Introduction

Type 2 diabetes (T2D) is now recognised as a pandemic-level public health concern, as evidenced by the increase in the number of patients with T2D from approximately 200 million in 1990 to over 800 million by 2022 [1]. T2D is associated with microvascular and macrovascular complications but also increases the risk of certain cancers, including colorectal cancer (CRC). Epidemiological studies indicate that the incidence of CRC is approximately 20–30% higher in patients with T2D compared to those without T2D [2]. Moreover, T2D has been shown to hurt cancer prognosis; for example, CRC patients with pre-existing T2D show poorer long-term survival than patients without T2D [2]. Therefore, identifying a biomarker linking diabetes to pre-cancer lesions is of great clinical and research importance.

Chronic inflammation is a key link between T2D and its related complications. T2D is now recognised as a pro-inflammatory condition that contributes to insulin resistance and tissue damage [3]. In this context, galectin-3 (Gal-3)—a β-galactoside-binding lectin secreted by macrophages and other cells—may be considered a potential biomarker connecting metabolic dysfunction with inflammatory responses [4]. Gal-3 has been implicated in regulating glucose metabolism, fat accumulation, and immune activation [4]. Serum Gal-3 levels were significantly higher in T2D patients than those in healthy controls and showed positive correlations with glycated haemoglobin (HbA1c) and C-reactive protein, linking Gal-3 to poor glycaemic control and systemic inflammation [5]. Gal-3 has also been associated with the development of T2D complications. Recent clinical studies report that T2D patients with advanced microvascular complications (such as nephropathy) tend to have higher serum Gal-3 concentrations than patient groups without such complications [6]. Another study has shown that Gal-3 can serve as a prognostic marker for cardiovascular events and heart failure in individuals with metabolic risk profiles, including those with T2D [3].

Recent studies suggest that Gal-3 plays a key role in tumour biology. Gal-3 can promote processes such as tumour cell adhesion, invasion, evasion of apoptosis, and immune suppression, thereby facilitating tumour progression and metastasis [7]. In CRC, higher serum Gal-3 concentrations were observed in patients with more advanced TNM stages and Duke’s stages, as well as higher carcinoembryonic antigen (CEA) levels and venous invasion [8]. Elevated Gal-3 was also associated with the presence of metastasis and poorer prognosis [9,10,11,12]. However, Gal-3 expression was not significantly correlated with gender, age, the degree of pathological differentiation, tumour location or size, or the presence of distant or lymph node metastases [8]. While the relationship between Gal-3 and colorectal polyps (CRPs) has not yet been fully explored, it is well-established that 95% of colorectal cancers arise from adenomatous polyps. This process follows the so-called adenoma–carcinoma sequence, which describes the gradual accumulation of genetic mutations (including *APC*, *KRAS*, *TP53*) leading from normal mucosa to precursor lesions (CRPs) and eventually to malignant tumours in approximately 10–15 years [13]. The second mechanism of colorectal carcinogenesis is the so-called serrated pathway, which involves hyperplastic serrated CRP, traditional serrated adenomas, and sessile serrated adenomas. These lesions may also undergo malignant transformation, primarily through mechanisms involving microsatellite instability. They are estimated to account for approximately 15–30% of CRC cases [14].

Malignant potential is present in all adenomas, and risk is determined according to some features of individual colorectal polyps (CRPs) [15], like diameter greater than 10 mm, histology type (villous), and high-grade dysplasia. Given the risk of malignancy, all identified CRPs should be endoscopically resected and subjected to histopathological evaluation. The removal of premalignant lesions has been demonstrated to significantly reduce both the incidence and mortality of colorectal cancer (CRC) [14]. Colonoscopy remains the gold standard modality for both diagnosis and therapeutic intervention [16].

Our study aimed to investigate the association between serum Gal-3 levels and insulin resistance and to explore whether this relationship contributes to the increased prevalence of CRPs in patients with T2D. Furthermore, we wanted to evaluate whether Gal-3 holds promise as a prognostic biomarker in this context.

## 2. Results

### 2.1. Study Groups

A total of 80 patients were analysed: 36 patients with T2D (T2D+) and 44 patients without T2D (T2D−). The mean age of all study participants was 63 years (9.9 ± SD), and the mean body mass index (BMI) was 28.7 kg/m^2^ (5.4 ± SD). Women constituted 58.75% of patients. There are differences between the T2D+ and T2D− groups regarding age, BMI, waist-to-height ratio (WHTR), and being an ex-smoker. The mean duration of T2D was 13 years, ranging from 1 to 39 years. The mean HbA1c in the T2D+ group was 7.47% (58 mmol/mol). One-third of T2D+ patients had at least one vascular diabetes complication. Almost half of the patients with diabetes were treated with metformin alone (47%), 42% with metformin and insulin, 5% with sulfonylurea only, 3% with an SGLT-2 inhibitor only, and 3% with diet only.

In contrast, the CRP+ (41 patients) and CRP− (39 patients) groups were comparable regarding socio-demographics. A total of 57.5% are women, with a mean age of 64 years, a BMI of 29 kg/m^2^, and a mean WHTR of 0.6 m.

### 2.2. Colonoscopy Results

No abnormalities were discovered in 30% (n = 11) of T2D+ and 23% (n = 10) T2D- patients. More than one CRP was found in 19% of T2D+ and 27% of T2D− patients. Hyperplastic polyps were found in 8% (n = 3) of T2D+ and 18% (n = 8) of T2D− patients. Colorectal adenomas were found in 50% (n = 18) of T2D+ and 52% (n = 23) of T2D− patients. CRPs with dysplasia were found more frequently in patients with diabetes compared to those without diabetes: in 42% vs. 39% of cases for low-grade dysplasia and 8% vs. 2% of patients for high-grade dysplasia. Most cases of colorectal cancer in individuals with CRPs (5/6) were found in the T2D+ group.

### 2.3. Gal-3, Clinical Variables

The mean serum Gal-3 level in all patients was 13.63 ng/mL (±4.53 SD). In all patients, the serum Gal-3 level was higher in women than in men (13.63 vs. 11.80, *p* < 0.05), and it showed a statistically significant positive correlation with age (rho = 0.281; *p* = 0.012), gender (rho = 0.220; *p* = 0.049), serum peptide C levels (rho = 0.957; *p* = 0.006), and serum IGF-1 levels (rho = −0.417; *p* < 0.001, Supplementary Materials Figure S1) in all patients, and for patients without T2D (T2D-), it also showed a statistically significant positive correlation with fasting plasma glucose (rho = −0.406; *p* = 0.009) (Table 1).

**Figure 1:**
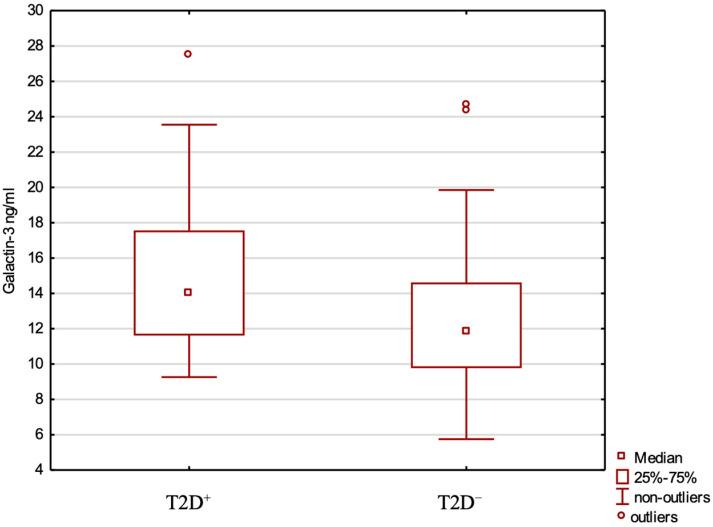
Galectin-3 levels in the T2D+ and T2D− groups.

Serum Gal-3 levels were significantly higher in T2D+ than T2D− patients (14.93 ng/mL vs. 12.57; *p* = 0.02) (Figure 1). In the T2D+ group, the median serum Gal-3 level in women was 17.21 (13.30–21.47) ng/mL, while in men, it was 12.08 (9.82–12.26) ng/mL. In the T2D+ group, Gal-3 concentration did not differ significantly between patients treated with metformin only (n = 16) and those treated with insulin ± another oral drug other than metformin (n = 15) (median 12.68, IQR 5.40 vs. median 11.37, IQR 8.22, *p* = 0.363). There is no relationship between serum Gal-3 and lipid-lowering therapy (Table 1).

**Figure 2:**
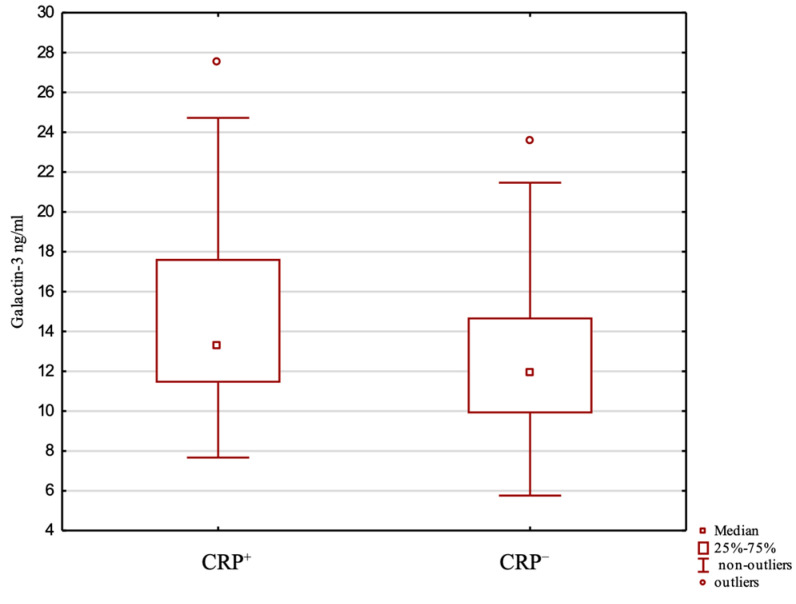
Galectin-3 levels for CRP+ and CRP− patients.

In the CRP + group, the mean serum Gal-3 level was not statistically significantly higher than that in the CRP− group (13.6 ng/mL (±4.35 SD) vs. 12.6 (±3.77 SD)) (Figure 2). There were no correlations of serum Gal-3 with anthropometric or laboratory variables for the CRP+ and CRP− groups.

Multiple regression analysis was used to assess factors influencing serum Gal-3 levels in all patients and the CRP+ group (Table 2). The standardised partial regression coefficient for body weight was −0.355 (*p* = 0.004) across the entire patient cohort.

An optimal Gal-3 cutoff value of ≥13.92 ng/mL for diabetes detection was established, corresponding to a sensitivity of 52.78% and specificity of 70.45% (AUC = 0.643; 95% confidence interval, 0.523–0.764). The two logistic regression models with serum Gal-3 level and, in the first case, classical risk factors for T2D development and, in the second case, age, BMI, HDL, CH, API, and FPG did not confirm that the Gal-3 level is an independent risk factor of T2D (Table 3).

The optimal cutoff value for detecting CRPs was a Gal-3 level ≥ 13.92 ng/mL, which corresponded to a sensitivity of 34.15% and a specificity of 89.74% (AUC = 0.623; 95% CI, 0.498–0.749). Logistic regression analysis identified age, body weight, API, and Gal-3 as independent variables significantly associated with CRPs (Table 4). T2D was not a significant predictor in this model.

## 3. Discussion

To the best of our knowledge, this is the first study to assess serum Gal-3 concentrations in patients with CRPs. Our findings suggest that elevated serum Gal-3 levels (≥13.92 ng/mL) might be a candidate biomarker of CRP/cancer development, particularly in the presence of other established factors such as age, body weight, and abdominal obesity, rather than as a standalone discriminator. Additionally, we observed significant positive correlations between Gal-3 levels and age, gender, and peptide C levels and negative correlations with FPG and IGF-1. These results are consistent with those of previous studies. De Boer’s study in the general population reported that Gal-3 levels varied with gender and age [15], while Ho’s study found a positive association with age but no relationship with gender [16]. Other research has also confirmed a correlation between Gal-3 and body weight [15,16,17]. Altun observed a positive correlation between Gal-3 levels, BMI (comparing obese and non-obese patients), and CH levels, but found no correlation between Gal-3 and insulin or HOMA-IR [18].

Moreover, both our study and previous research have reported significantly elevated galectin-3 (Gal-3) levels in patients with type 2 diabetes (T2D) compared to individuals without T2D. Gal-3 has been implicated in the development of insulin resistance [19]. In a study by Yilmaz involving 57 diabetic patients, Gal-3 levels were found to correlate with FPG and HOMA-IR, and the protein was identified as a predictor of T2D, even after adjusting for age, BMI, HOMA-IR, and HDL and TG levels [20]. Similarly, in a cohort of 20 diabetic patients, Gal-3 levels showed significant correlations with fasting insulin, HOMA-IR, and the insulin sensitivity index [21]. Zhang also reported an independent association between Gal-3 levels and HOMA-IR [22]. In a study involving 30 patients with T2D, no correlation was found between Gal-3 and BMI, WHTR, FPG, LDL, HDL, or CH; however, Gal-3 levels were negatively correlated with HbA1c [23], a finding that was also reported in studies by Bobronnikova and Seferovic [24,25].

For several years, research has focused on the relationship between Gal-3 levels and carcinogenesis. Our study underscores a potential association between Gal-3 and pre-cancerous CRPs. The literature suggests a possible link between Gal-3 and CRC [8]. Higher Gal-3 concentrations in patients with CRPs have been associated with a greater degree of colonic wall invasion, poorer histological differentiation, and tumour progression [26]. Additionally, elevated Gal-3 levels were linked to shorter survival in CRC patients [27]. However, studies by Wu and Tsuboi found no significant correlation between Gal-3 levels and tumour staging in CRC [28,29]. A study examining both blood and faecal Gal-3 concentrations in 60 CRC patients demonstrated that higher faecal Gal-3 levels were associated with higher nuclear grade, poor tumour tissue differentiation, advanced TNM stage, and metastatic disease [30].

To enhance the clinical relevance of our findings, future studies should address several key issues. Longitudinal designs are needed to assess whether changes in circulating Gal-3 reflect disease progression or therapeutic effects, particularly in metformin-treated patients. Such approaches would help clarify causal relationships and evaluate Gal-3 as a dynamic biomarker in metabolic and inflammatory states. Mechanistic studies using Gal-3 knockout models or specific inhibitors are also essential to define its role in insulin resistance, inflammation, and fibrosis and to explore whether Gal-3 mediates the anti-inflammatory effects of metformin. Additionally, validation in larger, more diverse, ideally multicentre cohorts is necessary to confirm generalizability and account for confounders such as disease duration, glycaemic control, and comorbidities.

Although we found a statistically significant association between elevated Gal-3 levels and the presence of CRPs, the effect size was modest (AUC 0.623; 95% CI 0.520–0.727), limiting its standalone diagnostic value. Gal-3 is more likely to be clinically useful as part of a multi-analyte panel that reflects overlapping metabolic, inflammatory, and fibrotic pathways involved in colorectal carcinogenesis among patients with T2D. Future research should incorporate complementary biomarkers—such as high-sensitivity C-reactive protein, interleukin-6, the adiponectin/leptin ratio, and advanced glycation end products—and apply untargeted metabolomic or transcriptomic profiling to uncover novel mechanisms. The use of multivariate or machine learning models may further clarify the added predictive value of Gal-3 within integrated multi-omics frameworks, ultimately defining its role in personalised risk assessment.

### Strengths and Limitations of This Study

One of the strengths of this study is the complete clinical and biochemical characterisation of the subjects. However, the study population cannot be considered entirely homogeneous due to significant differences in BMI and age between patients with T2D and controls. Additionally, recruitment was prematurely halted due to the COVID-19 outbreak, thereby limiting access to elective procedures such as colonoscopy. This resulted in a necessarily limited sample size and the absence of longitudinal follow-up, both of which constrain the generalizability of our findings. The current findings are therefore to be interpreted as hypothesis-generating rather than definitive. Nevertheless, we believe that our observations will provide valuable preliminary data and might provoke further investigation in larger, prospective cohorts.

## 4. Materials and Methods

### 4.1. Study Design and Population

A cross-sectional study was conducted from February 2019 to March 2020 in the Central Teaching Clinical Hospital UCC MUW in Warsaw (Poland) on 80 consecutive patients undergoing colonoscopy. The study inclusion criteria were the following: age ≥ 18 years and no history of cancer in the last five years or chronic inflammatory bowel disease or clinically significant cardiovascular disease (acute coronary syndromes during the previous year, heart failure in NYHA class III–IV). The exclusion criteria included acute infection, chronic inflammation, C-reactive protein levels ≥ 10 mg/L, diabetes other than T2D, acromegaly, or CRC or colorectal surgery in the past.

All patients underwent comprehensive medical examinations, including anthropometric measurements. Fasting blood samples were obtained via venipuncture of the antecubital vein. Insulin resistance was estimated using the Homeostasis Model Assessment of Insulin Resistance (HOMA-IR) as described by Matthews et al. (1985) [31]. The patients were referred for colonoscopy for the following reasons: changes in bowel movement patterns (31%), anaemia (22%), and cancer screening (22%).

The patients were divided into two subgroups based on the presence of (1) type 2 diabetes (T2D^+^ vs. T2D^−^) and (2) colorectal polyps (CRP^+^ vs. CRP^−^). We did not stratify patients into four groups (i.e., T2D^+^/CRP^+^, T2D^+^/CRP^−^, T2D^−^/CRP^+^, T2D^−^/CRP^−^) due to the limited number of patients in each subgroup. The baseline characteristics of the study participants are presented in Table S1 of the Supplementary Materials.

The blood samples for Gal-3 measurement were centrifuged and stored at −70 °C until the assays were conducted. The serum levels of Gal-3 were quantified using a solid-phase enzyme-linked immunosorbent assay (ELISA) KIT (Catalogue Numbers BMS279-4 and BMS279-4TEN, Thermo Fisher, Vienna, Austria). This assay is highly sensitive (minimum detectable dose: 0.29 ng/mL) and shows no significant cross-reactivity or interference. All measurements were performed in duplicate. Calibration and standardisation were carried out following the manufacturer’s protocol. The inter- and intra-assay coefficients of variation were 7.5% and 5.4%, respectively.

This study was conducted according to the Guideline for Good Clinical Practice rules. The Committee on Bioethics approved this study at the Medical University of Warsaw, KB/42/2018 (18 April 2018), and all the patients gave their written informed consent to participate in this study.

### 4.2. Statistical Analysis

Continuous variables are presented as the mean ± standard deviation (SD) or median with the interquartile range (IQR), depending on their distribution. Categorical variables are reported as frequencies (numbers and percentages). The normality of the data was assessed using the Shapiro–Wilk test, which indicated a non-normal distribution for the relevant variables. As a result, the Mann–Whitney U-test was used to compare non-normally distributed continuous variables between groups, and the chi-square test was applied to categorical variables.

Spearman’s rank correlation was used to examine the relationships between serum Gal-3 levels and clinical or laboratory markers, as the data did not meet the normality assumptions required for Pearson correlation. The ordinal nature of some variables further supported the use of Spearman’s method. Correlation coefficients were calculated to assess the association between serum Gal-3 and other markers, as well as binary outcomes for T2D and CRP presence (yes/no).

Logistic regression analyses were conducted to investigate the relationship between clinical and laboratory markers and binary outcomes for T2D and CRPs. Relevant covariates, such as age, BMI, and lipid profile parameters, were included in the models to adjust for potential confounders. Odds ratios (ORs) with 95% confidence intervals (CIs) were reported.

Effect sizes, including ORs and their CIs, were reported alongside *p* values to offer a more comprehensive interpretation of the results. Statistical significance was defined as *p* < 0.05. All statistical analyses were performed using SPSS version 27 for logistic regression, while descriptive and inferential statistics were calculated using Statistica 13.3 (StatSoft, Krakow, Poland).

## 5. Conclusions

Serum Gal-3 levels are modulated by a range of clinical factors that contribute to the pathogenesis of various conditions, including metabolic disorders of carbohydrate and lipid metabolism, which play a key role in the progression of diabetes and colorectal cancer. The involvement of Gal-3 in these processes underscores its potential as a biomarker for the early detection of both cancerous and pre-cancerous colorectal lesions. Although not disease-specific, galectin-3 (Gal-3) serves as a marker of early pathological changes induced by metabolic alterations within the tumour microenvironment. Furthermore, as a prognostic indicator, elevated Gal-3 levels may signal tumour progression, metastasis, or recurrence through its role in cellular adhesion and angiogenesis. To comprehensively define Gal-3’s role in the pathogenesis of CRP, T2D, and CRC, larger cross-sectional and longitudinal studies are required.

## Figures and Tables

**Table 1:** Correlation coefficients between serum galectin-3 concentrations and other parameters.

Correlation of Gal-3 with	All Patients (N = 80)		CRP+ (N = 41)		CRP− (N = 39)		T2D+ (N = 36)		T2D− (N = 44)	
	Spearman Correlation	*p* Value	Spearman Correlation	*p* Value	Spearman Correlation	*p* Value	Spearman Correlation	*p* Value	Spearman Correlation	*p* Value
FPG (mg/dL)	0.201	0.740	0.191	0.232	0.252	0.122	0.286 *	0.112 *	−0.406 *	0.009 *
CH (mg/dL)	−0.142	0.208	−0.319	0.042	0.006	0.970	0.121 *	0.509 *	−0.210 *	0.194 *
TG (mg/dL)	0.060	0.594	0.030	0.852	0.107	0.518	−0.011 *	0.954 *	0.075 *	0.646 *
HDL (mg/dL)	−0.184	0.102	−0.177	0.267	−0.192	0.242	−0.093 *	0.613 *	−0.296 *	0.064 *
HOMA-IR	−0.117	0.303	−0.016	0.919	−0.192	0.241	−0.315 *	0.079 *	−0.119 *	0.463 *
API	0.141	0.211	0.116	0.470	0.168	0.305	0.015 *	0.936 *	0.211 *	0.192 *
Fasting insulin (uIu/mL)	−0.171	0.128	−0.068	0.671	−0.232	0.155	−0.301 *	0.094 *	−0.137 *	0.399 *
PEPTIDE C (ng/mL)	0.957	0.006	0.048	0.764	−0.043	0.796	−0.313 *	0.081 *	0.100 *	0.537 *
IGF-1 (ng/mL)	−0.417	<0.001	−0.450	0.003	−0.433	0.006	−0.342 *	0.055 *	0.319 *	0.045 *
SEX	0.220	0.049	0.326	0.037	0.102	0.537	0.531 *	0.020 *	0.110 *	0.944 *
AGE (years)	0.281	0.012	0.311	0.048	0.249	0.127	- *	- *	- *	- *
Body weight	−0.010	0.931	−0.018	0.910	−0.038	0.818	- *	- *	- *	- *
BMI (kg/m^2^)	0.128	0.259	0.210	0.188	0.020	0.991	- *	- *	- *	- *
WHTR	0.158	0.161	0.193	0.226	0.086	0.602	- *	- *	- *	- *
Lipid-lowering therapy	−0.058	0.610	−0.111	0.491	0.066	0.688	−0.230	0.205	−0.189	0.267
Duration of T2D (years)			0.750 *	0.794 *	0.183 *	0.532 *	0.160 *	0.383 *		
Duration of metformin (years)			0.272 *	0.347 *	0.393 *	0.164 *	0.234 *	0.197 *		
Duration of insulin (years)			0.124 *	0.673 *	0.317 *	0.270 *	0.157 *	0.384 *		
Dose of insulin (IU/d)			−0.096 *	0.744 *	0.411 *	0.144 *	0.159 *	0.384 *		

T2D, type 2 diabetes; CRPs, colorectal polyps; FPG, fasting plasma glucose; CH, total cholesterol; TG, triglyceride; HDL, high-density lipoprotein cholesterol; HOMA-IR, homeostatic model assessment for insulin resistance; API, atherogenic index of plasma; IGF-1, insulin-like growth factor 1; BMI, body mass index; WHTR, waist-to-height ratio; IU/d, international units per day. * Adjusted for the following confounders: age, BMI, WHTR, smoking status.

**Table 2:** Multiple regression analysis to examine influencing factors of galectin-3.

	All Patients, N = 80 (R^2^ = 0.3; R = 0.678)				CRP+, N = 41 (R^2^ = 0.011; R = 0.584)			
	Standardised Coefficients	Partial Correlation	t	*p* Value	Standardised Coefficients	Partial Correlation	t	*p* Value
AGE	−0.05	−0.04	−0.31	0.75	0.02	0.01	0.07	0.94
BMI	1.00	0.29	2.37	0.21	0.34	0.20	0.67	0.51
WHTR	0.16	0.09	0.69	0.50	0.20	8.25	0.29	0.78
Body weight	−1.21	−0.36	−2.97	0.00	−0.07	−0.02	−0.11	0.91
Duration of T2D	0.20	0.12	0.93	0.36	0.39	0.17	1.68	0.10
FPG	0.01	0.01	0.05	0.96	−0.15	−0.01	−0.82	0.42
CH	−0.20	−0.19	−1.50	0.14	0.12	0.01	0.55	0.9
TG	0.37	0.10	0.82	0.42	−0.15	−0.01	−0.29	0.77
HDL	−0.36	−0.13	−1.04	0.31	0.04	0.01	0.11	0.91
HOMA-IR	0.56	0.08	0.61	0.54	−1.31	−4.69	−1.40	0.17
AIP	−0.54	−0.10	−0.82	0.42	0.11	1.69	0.17	0.87
Fasting insulin	−0.75	−0.11	−0.87	0.39	0.63	0.72	0.72	0.48
PEPTIDE C	0.18	0.13	0.99	0.32	0.41	1.89	1.57	0.13
IGF-1	−0.16	−0.16	−1.30	0.20	−0.03	−0.00	−0.14	0.89

CRPs, colorectal polyps; BMI, body mass index; WHTR, waist-to-height ratio; T2D, type 2 diabetes; FPG, fasting plasma glucose; CH, total cholesterol; TG, triglyceride; HDL, high-density lipoprotein cholesterol; HOMA-IR, homeostatic model assessment for insulin resistance; API, atherogenic index of plasma; IGF-1, insulin-like growth factor 1.

**Table 3:** Risk of diabetes—logistic regression analysis.

	Variable	*p* Value	OR	CI −95%	CI +95%
Model 1.	Gender	0.62	0.72	0.20	2.64
	Age	0.00	1.13	1.05	1.21
	BMI	0.02	1.14	1.02	1.27
	HOMA-IR	0.77	0.95	0.69	1.32
	TG	0.03	1.02	1.00	1.03
	HDL	0.09	0.96	0.91	1.01
	Gal-3	0.31	1.08	0.93	1.26
	For the model: *p* < 0.001; Nagelkerke R-square 0.557, −2 log-likelihood 67.025, Hosmer–Lemeshow test: *p* 0.928				
Model 2.	Age	0.02	1.12	1.02	1.24
	BMI	0.02	1.20	1.03	1.40
	HOMA-IR	0.13	0.65	0.37	1.14
	HDL	0.02	1.19	1.03	1.38
	CH	0.01	0.95	0.91	0.99
	API	0.01	98,504	21.8	444,516,456
	FPG	0.01	1.07	1.02	1.12
	Gal-3	0.58	1.06	0.88	1.27
	For the model: *p* < 0.001; Nagelkerke R-square 0.557, −2 log-likelihood 67.025, Hosmer–Lemeshow test: *p* 0.928				

OR, odds ratio; CI, confidence interval; BMI, body mass index; HOMA-IR, homeostatic model assessment for insulin resistance; TG, triglyceride; HDL, high-density lipoprotein cholesterol; API, atherogenic index of plasma; FPG, fasting plasma glucose; CH, total cholesterol.

**Table 4:** Risk of polyps—logistic regression analysis.

	*p* Value	OR	CI −95%	CI +95%
Sex	0.291	2.301	0.490	10.819
Age	0.003	1.116	1.038	1.201
Body weight	0.016	1.105	1.019	1.199
Waist circumference	0.153	0.941	0.865	1.023
T2D	0.156	0.350	0.082	1.491
HOMA-IR	0.059	0.049	0.002	1.122
Fasting insulin	0.107	2.002	0.061	4.658
API	0.007	2.743	1.314	5.728
IGF-1	0.059	1.018	0.999	1.037
CH	0.792	0.998	0.982	1.014
Gal-3	0.031	1.178	1.015	1.366

For the model: *p* < 0.001; Nagelkerke R-square 0.453, −2 log-likelihood 77.627, Hosmer–Lemeshow test: *p* 0.471. OR, odds ratio; CI, confidence interval; T2D, type 2 diabetes; HOMA-IR, homeostatic model assessment for insulin resistance; API, atherogenic index of plasma; IGF-1, insulin-like growth factor 1; CH, total cholesterol.

## Data Availability

Data are available from the authors on reasonable request.

## Supplementary Materials

The supporting information can be downloaded at https://www.mdpi.com/article/10.3390/ijms26167662/s1.

## Abbreviations

The following abbreviations are used in this manuscript:

T2D	type 2 diabetes
CRC	colorectal cancer
Gal-3	galectin-3
HbA1c	glycated haemoglobin
CRPs	colorectal polyps
BMI	body mass index
SD	standard deviation
OR	odds ratio
CI	confidence interval
WHTR	waist-to-height ratio
FPG	fasting plasma glucose
IGF-1	insulin-like growth factor 1
HOMA-IR	homeostatic model assessment for insulin resistance
AIP	atherogenic index of plasma
CH	total cholesterol
TGs	triglycerides
HDL	high-density lipoprotein cholesterol
LDL	low-density lipoprotein cholesterol
