# Supplementary material for: Cross-Sectional Study of Serum Galectin-3 Levels in Patients with Type 2 Diabetes and Colorectal Polyps
Source: Int J Mol Sci. 2025 Aug 8;26(16):7662. doi: 10.3390/ijms26167662 (PMC12386645; doi:10.3390/ijms26167662)
Supplement: Supplementary file 1 [file ijms-26-07662-s001.zip › ijms-3773707-supplementary.pdf]

Table S1. The patients' characteristics according to the presence of type 2 diabetes mellitus and colorectal polyps.

| | DM2+ (N=36) | DM2- (N=44) | P VALUE | CRP + (N=43) | CRP- (N=37) | P VALUE |
|---|---|---|---|---|---|---|
| AGE (years) (mean±sd) | 68.3 (1.70) | 59,2 (1,99) | .001 | 65.8 (10.8) | 62.5 (11.6) | .237 |
| WOMEN (n) | 19 | 28 | .326 | 24 | 22 | .742 |
| BMI (kg/m2) (mean±sd) | 31.7 (1.36) | 26,24 (0.81) | .002 | 28.6 (7.23) | 29.0 (7.26) | .870 |
| WHtR | 0.63 (0.02) | 0.55 (0.013) | .000 | 0.58 (0.11) | 0.59 (0.09) | .540 |
| NEVER SMOKED (n) | 10 | 18 | .221 | 12 | 14 | .344 |
| EX-SMOKER (n) | 21 | 15 | .030 | 19 | 23 | .486 |
| EX-PACK YEAR | 12.7 (3.39) | 5.36 (1,89) | .018 | 7.17 (11.3) | 7.93 (17.8) | .802 |
| ACTIVE SMOKER (n) | 4 | 10 | .174 | 9 | 5 | .384 |
| PACK YEAR | 2.80 (1.43) | 6.24 (2.17) | .177 | 5.56 (13.4) | 3.67 (10.7) | .414 |
| OCCASIONAL ALCOHOL CONSUMPTION (N)* | 16 | 26 | .192 | 23 | 19 | .849 |
| MODERATE REGULAR PHYSICAL ACTIVITY (n) | 13 | 17 | .816 | 14 | 14 | .622 |
| FAMILY HISTORY OF DM (n) | 22 | 15 | .016 | 17 | 20 | .194 |
| FAMILY HISTORY OF CANCER (n) | 20 | 22 | .621 | 24 | 17 | .379 |
| FAMILY HISTORY OF CRC (n) | 3 | 9 | .131 | 9 | 3 | .109 |
| LIPID-LOWERING THERAPY (n) | 22 | 5 | .000 | 15 | 14 | .784 |
| DM | 36 | 44 | X | 20 | 18 | .849 |
| POLYP (n) | 18 | 23 | .840 | 43 | 0 | X |
| FPG (mg/dl) | 155 (12.2) | 93.2 (2.43) | .000 | 122 (61.7) | 122 (56.2) | .685 |
| CH (mg/dl) | 148 (5.35) | 185 (8.12) | .000 | 175 (54.5) | 163 (44.8) | .499 |
| TG (mg/dl) | 158 (12.0) | 110 (6.97) | .000 | 144 (89.0) | 123 (57.2) | .309 |
| HDL (mg/dl) | 45.1 (2.02) | 55.7 (2.54) | .003 | 48.1 (14.7) | 52.7 (15.9) | .213 |
| HOMA-IR | 3.05 (0.51) | 1.94 (0.19) | .081 | 1.95 (1.21) | 2.93 (3.01) | .288 |
| AIP | 0.52 (0.42) | 0.28 (0.33) | .000 | 0.44 (0.27) | 0.35 (0.26) | .222 |
| HBA1c% | 7.47 (0.28) | X | X | 7.31 (1.60) | 7.07 (1.80) | .409 |
| INSULIN (uIU/ml) | 10.5 (1.62) | 7.48 (0.67) | .200 | 7.25 (3.80) | 10.4 (9.80) | .309 |
| PEPTIDE C (ng/ml) | 2.52 (0.20) | 2.03 (0.12) | .073 | 2.22 (0.94) | 2.27 (1.11) | .919 |
| IGF-1 (ng/ml) | 134 (7.93) | 141 (6.32) | .542 | 138 (42.2) | 133 (41.6) | .521 |

FPG fasting plasma glucose, CH total cholesterol, TG triglycerides, HOMA-IR homeostatic model assessment for insulin resistance, API atherogenic index of plasma, BMI body mass index, WHtR waist to height ratio, DM2 diabetes mellitus, CRP colorectal polyp

* drinking alcohol less than once a month - for men: consuming no more than 6 standard portions at a time (60 g of 100% alcohol), i.e. no more than 3 half-liter bottles of beer, 3 glasses of wine with a capacity of 200 ml or 180 ml of vodka; for women: consuming no more than 4 standard portions at a time (40 g of 100% alcohol), i.e. no more than two half-liter bottles of beer, 2 glasses of wine with a capacity of 200 ml each or 120 ml of vodka.

Figure S1. Correlation of serum Gal-3 levels with IGF-1 levels in all patients (r=-0.367).

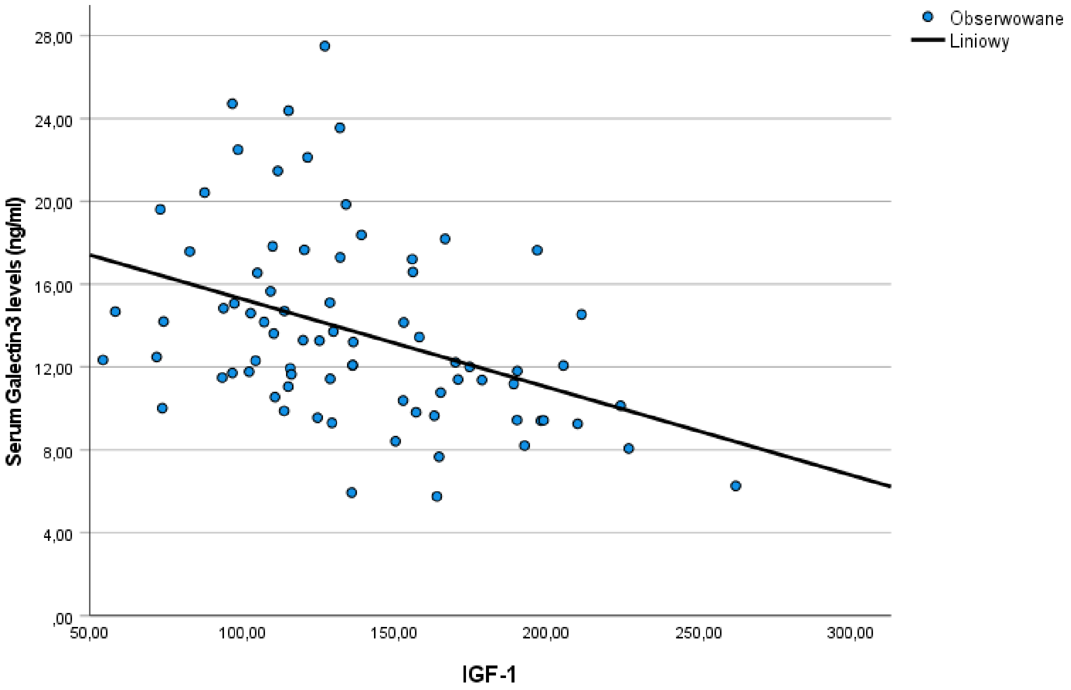
Obserwowane
Liniowy
Serum Galectin-3 levels (ng/ml)
28,00
24,00
20,00
16,00
12,00
8,00
4,00
,00
50,00
100,00
150,00
200,00
250,00
300,00
IGF-1
